# A Comparison of the Chicken and Turkey Proteomes and Phosphoproteomes in the Development of Poultry-Specific Immuno-Metabolism Kinome Peptide Arrays

**DOI:** 10.3389/fvets.2014.00022

**Published:** 2014-11-13

**Authors:** Ryan J. Arsenault, Brett Trost, Michael H. Kogut

**Affiliations:** ^1^United States Department of Agriculture, Southern Plains Agricultural Research Center (SPARC), Agricultural Research Service, College Station, TX, USA; ^2^Department of Computer Science, University of Saskatchewan, Saskatoon, SK, Canada

**Keywords:** kinome, chicken, turkey, poultry, peptide array, phosphorylation, proteome, immuno-metabolism

## Abstract

The use of species-specific peptide arrays for the study of animal kinomes has a proven track record of success. This technique has been used in a variety of species for the study of host–pathogen interactions and metabolism. Species-specific peptide arrays have been designed previously for use with chicken but a turkey array has never been attempted. In addition, arrays designed around individual cellular functions have been designed and utilized, but cross-function immuno-metabolic arrays have not been considered previously. Antecedent to designing separate chicken and turkey immuno-metabolic kinome peptide arrays, we show that while the chicken and turkey genomes are quite similar, the two species are much more distinct at the proteome and phosphoproteome levels. Despite a genome identity of approximately 90%, we observe that only 83% of chicken and turkey orthologous proteins display sequence matches between the two species. Further, less than 70% of kinase recognition target sequences are exact matches between chicken and turkey. Thus, our analysis shows that, at the proteome and kinome level, these two species must be considered separately in the design of novel peptide arrays. Our ultimate array design covers numerous immune and metabolic processes including innate and adaptive immunity, inflammatory responses, carbohydrate, protein, and fat metabolism, and response to hormones. We have shown the proteomic and phosphoproteomic diversity of chicken and turkey and have designed a valuable research tool for the study of immuno-metabolism within these two species.

## Introduction

Genomics and genetics are dominant approaches in the study of physiology and disease states across species. The tools within these fields have advanced substantially and enable the rapid collection of significant amounts of data; this now includes the rapid sequencing of entire species genomes (1). While sequencing the complete genome of an organism is an important means of biological understanding and discovery, it can be very difficult to translate this information into individual host responses due to stimulation or treatment, and ultimate organism phenotype. Transcriptomics, being a more focused branch of genetics, allows one to consider only the expressed genes converted to mRNA. There are a wide variety of tools available for numerous species to study the transcriptome. The downside of transcriptomic approaches, especially when attempting to determine final phenotype, is that there are several processes and potential disruptions that can occur before the final active protein is generated. These include gene silencing, mRNA stability, translation, translational efficiencies, protein turnover, sequestration of enzymes from substrates, and the multitude of post-translation modifications, of which phosphorylation is a major class.

Studying physiology and organism response at the proteome-level allows one to consider biology near or at the final phenotype. The active proteins are the ultimate effectors that influence phenotype. Within proteomics studying the enzymatic activity of a protein allows one to consider the final step in a cellular response. Kinomics is the study of kinase enzymes activated under given conditions within a cell or tissue. Kinases are enzymes that phosphorylate proteins, changing the target protein’s activity in some way (2). Cellular signal transduction through protein phosphorylation is a post-translational modification that plays a role in the regulation of nearly every cellular process and function (3); thus, the study of kinomics can provide insight into the various cellular processes at an active protein level. In this type of approach, all of the intervening steps between gene and phenotype, such as gene silencing, the effects of non-coding RNA, and improper protein folding and function, are eliminated.

The technique described here to study the cellular kinome is peptide microarrays. There are other techniques that have been used to study the kinome including phospho-antibodies and mass-spectrometry (4, 5). Peptide arrays have a number of advantages including the availability of reagents and equipment, the ability to focus only on the active kinases within a sample, and the ability to tailor the analysis to particular species, cellular functions, and individual phosphorylation sites (6). Peptide arrays have been used to study the kinomics of cellular biology (7, 8) and host–pathogen interactions (9–13). One of the key peptide array technology developments was the establishment of species-specific peptide arrays, providing a tool for the study of veterinary species at the kinome level (14).

A species-specific peptide array allows for the study of the kinome of nearly any species. The process of designing a species-specific array begins with the known phosphorylation sites within the species of interest, as well as a database of phosphorylation sites from an evolutionarily close species (14). While some species such as human, mouse, rat, and fruit fly have relatively well-annotated proteomes and phosphoproteomes, other species such as chicken, turkey, sheep, and honey-bee are less well-studied. Within the custom-designed software pipeline DAPPLE (15), one uses databases of experimentally determined phosphorylation sites from other organisms to query the complete proteome of the species of interest for orthologous phosphorylation sites. Using DAPPLE, it is possible to determine if a kinase target sequence from the query species is present and located within the orthologous protein of the target species. Using these orthologous kinase target peptides, it is possible to design a species-specific kinome peptide array. This technique has been used to design species-specific peptide arrays for species as diverse as cow (9, 11), chicken (10), pig (16), and honey bee (17) for cellular functions from innate immunity (18) to metabolism (10). The primary advantage of using species-specific arrays is one does not have to rely on potential cross-reactions between a commercially available peptide array (usually human or mouse) and the species of interest. The kinase target sites printed onto the array are designed to be exact matches to the proteome of the given species. The limited homology in human peptide arrays for the study of other species (19) as well as the lack of cross-reactivity or unexpected cross-reactivity observed in species-specific antibodies (20) illustrates the fact that one cannot rely on cross-species cross-reactivity at the level of peptide recognition. Our own analysis has shown that the species variation is significantly greater at the phosphorylation target site sequence than at the genome level (6). Here, we show that this is true when comparing chicken and turkey phosphorylation target sequences as well.

The chicken (G*allus gallus)* and turkey (*Meleagris gallopavo*) diverged approximately 40 million years ago (21). Despite this length of separate evolution, the genomes of chicken and turkey are relatively similar. As a comparison, rhesus macaque and humans separated 25 million years ago, and these species display significantly greater genome differences than chicken and turkey (21). An Ensembl LastZ alignment between the human and macaque genomes show an 80% complete genome coverage, while the chicken and turkey genomes show an 89% complete genome coverage. Other avian genomes align significantly as well; a three-way alignment between chicken, turkey, and zebra finch shows alignments of 91.92, 92.39, and 81.51%, respectively (21).

Though the genomes of chicken and turkey appear relatively similar, more specific genetic study reveals evidence of why we cannot simply assume orthology. In the case of the immune systems of chickens and turkeys, species diversity is a key consideration. Genomic evidence involving the innate immune system points to stronger positive selection on these genes between chicken and turkey than between other species pairs including chicken-zebra finch and turkey-zebra finch (21). When comparing chicken to turkey, the level of positive selection of the innate immune genes (as measured by dN/dS ratio) approaches the level of non-immune genes (21). This level of positive selection would not be expected in a set of genes normally undergoing purifying selection. This result indicates that innate immune genes of these two species will be more evolutionarily divergent than would be anticipated, further strengthening the argument for a species-specific approach. Here, we have taken this comparative approach to the level of the proteome and phosphoproteome to understand the level of divergence between the chicken and turkey within the key physiological processes of immunity and metabolism.

The interface of the immune system and metabolism is an emerging field of study (22). The initial impetus for researchers to consider an immuno-metabolic perspective was the human health concerns related to obesity, diabetes, and metabolic disorder. Excessive fat deposition can lead to an innate immune inflammatory response. This chronic low-grade inflammation was linked to resultant diseases such as type 2 diabetes, fatty liver disease, and cirrhosis (22). Later, some of the classical metabolic energy sensors and energy switches, such as AKT1-3, AMPK, mTOR, and LKB1, were shown to be linked to CD8^+^ T cell functions (23). From there, links between metabolism, immunity, and host response to infectious disease grew. Within animal agriculture, and poultry production specifically, a consideration of the immuno-metabolic consequences will be invaluable. A focus solely on maximizing animal growth can reduce immune potential, while a strong immune response has negative consequences on growth (24). A consideration of the integrated whole may allow one to maximize growth and animal production without having a detrimental impact on animal health and immune potential. In this study, we show the nearly innumerable links between cellular signaling proteins classically characterized as members of either the immune or metabolic functional groups. Due to these links, we feel an integrated immuno-metabolic approach would be a valuable research perspective; thus, we describe here for the first time the design of chicken and turkey species-specific, immuno-metabolic peptide arrays for kinome analysis, based on the proteomic analysis of these two species.

## Materials and Methods

### Identification of putative chicken and turkey phosphorylation sites

The DAPPLE (15)^1^ software pipeline was used to identify phosphorylation sites in the chicken and turkey proteomes that were orthologous to experimentally determined phosphorylation sites from other organisms. The PhosphoSitePlus database (25)^2^ was used as the source of experimentally determined phosphorylation sites. At the time of analysis, it contained 229,173 such sites, most of which were from human, mouse, rat. Each of these sites was represented as a 15-mer kinase target sequence, with the potential phosphorylation residue in the center and seven residues on either side. In cases where the phosphorylation residue was too close to the N- or C-terminus of the full protein for this to be possible, the phosphorylation site was represented by the first 15 residues or last 15 residues of the full protein, respectively. For each of these peptides, DAPPLE performed a protein BLAST search using the peptide as the query, and the chicken or turkey proteome as the database. DAPPLE then reports the best match in the chicken or turkey proteome for that 15-mer peptide, as well as additional information that facilitates the selection of peptides to include on an array (for example of DAPPLE output see Table S2 in Supplementary Material).

### Peptide selection and array design

Once the list of putative chicken and turkey phosphorylation target sites were generated by DAPPLE, the individual peptides containing phosphorylation sites were selected for incorporation into the final array. This selection was based both on the design goals of the array, and on the confidence that a given chicken or turkey site identified by DAPPLE was a true phosphorylation site. With respect to the design goals of the array, the intention was to design an immuno-metabolic peptide array. As such, peptides were selected that derived from proteins falling into one of three categories: (1) proteins that could be considered central cellular signaling hubs (for example, AKT, MAPK, PI3K); (2) proteins involved in the innate and adaptive immune systems; and (3) proteins involved in metabolic processes (for example, glycolysis, fatty acid synthesis, protein catabolism, protein synthesis). With respect to the confidence that a given peptide identified by DAPPLE indeed contained a phosphorylation site, several criteria were considered. First, DAPPLE outputs the number of sequence differences between the query 15-mer and its best match in the chicken or turkey proteome. Peptides for which the number of sequences differences was small were preferentially selected for inclusion on the array. Second, DAPPLE determines whether the full protein corresponding to the query peptide, and the full protein corresponding to the query peptide’s closest match in the chicken or turkey proteome, are reciprocal BLAST hits (and thus are likely to be orthologs). We preferentially selected putative chicken or turkey phosphorylation sites for which the corresponding proteins were reciprocal BLAST hits. Third, DAPPLE outputs the phosphorylated residue in the corresponding full protein (e.g., Y25) for both the query phosphorylation site and its best hit. Putative chicken or turkey sites for which the position of the phosphorylated residue was similar to the query site were preferred.

### Characterization of proteome-level conservation between chicken and turkey

To determine the level of conservation between chicken and turkey at the proteome level, a list of orthologous proteins between the two species was downloaded from OrthoDB (26)^3^. For each pair of orthologs, the EMBOSS (27) program *needle* (28) was used to determine the optimal global alignment between the two proteins, as well as the % identity and the percentage of alignment positions that contained gaps.

### Characterization of kinase target sequence conservation between human and other species

The PhosphoSitePlus database (25) was filtered to include only phosphorylation sites from human. Using DAPPLE, the 15-mer peptides corresponding to these sites were used as BLAST queries against four target proteomes (chimpanzee, mouse, chicken, and turkey). The number of sequence differences between a given query peptide and its best match in the target proteome was recorded.

### Characterization of kinase target site conservation between chicken and turkey

Because few phosphorylation sites have been experimentally characterized in chicken, and none are known in turkey, it is difficult to directly assess the level of phosphorylation site conservation between these species. As such, the following procedure was performed to estimate the level of kinase target site 15-mer conservation. Using DAPPLE, the 15-mer peptide corresponding to each of the known phosphorylation sites in the entire PhosphoSitePlus database (25) was searched against the chicken proteome. All of the matches in the chicken proteome that had seven or fewer sequence differences from the known 15-mer peptide sequence were then used as input to DAPPLE, this time with the turkey proteome as the target. The number of sequence differences between a given chicken sequence, and its best match in the turkey proteome, was then recorded. This process was then repeated in reverse, with the contents of PhosphoSitePlus initially being searched against the turkey proteome, and then the matches with seven or fewer sequence differences being searched against the chicken proteome.

## Results

At the genome and gene level, the chicken and turkey show significant similarity. Genome coverage between the two species is 89%, while exon coverage is 90% (Ensembl LastZ alignment). Coverage represents the percentage of bases in the shorter of the input sequences that align. Pecan alignment of the chicken, turkey, and zebra finch genomes shows that chicken and turkey align at 91.92 and 92.39%, respectively (21). However, this level of genetic similarity does not translate to the protein level. The online database OrthoDB (26) lists 9,816 putative orthologs between chicken and turkey. As the chicken proteome contains approximately 23,000 proteins and the turkey proteome contains approximately 16,000 proteins (29), both chicken and turkey encode many proteins for which an ortholog does not exist in the other species. With respect to the proteins that do have orthologs in the other species, the average percent identity between these pairs of orthologs was 83.3%, with gaps in the alignment accounting for 12.4% of the difference (Table 1). As such, the level of similarity between chicken and turkey was lower at the protein level than at the genome level, and it is worth recalling that this level of difference is in the proteins that have been determined to be orthologous.

**Table 1:** **Comparison of chicken to turkey protein sequence identity**.

	% Sequence identity	% Due to sequence gaps	Total %	No. of protein pairs
Ortholog-to-ortholog comparison	83.28	12.37	95.65	9816

*Ortholog-to-ortholog comparisons are only those proteins shown to be orthologous based on OrthoDB (26). % Sequence identity indicates sequence similarity between pairs of proteins and % sequence gaps is the % of the protein that failed to align to due gaps in the sequence as opposed to amino acid mismatches*.

In the design of species-specific peptide arrays, the sequences of interest between orthologous proteins is the kinase target recognition sequence. These are sequences of 15 amino acids containing either a serine, threonine, or tyrosine central residue that is recognized by a specific kinase and phosphorylated (30). As a first step in analyzing the kinase target 15-mers, we utilized the large and well-annotated PhosphoSitePlus online database (25). At the time of analysis, this database contained 229,173 such phosphorylation target sequences. To determine the level of conservation of phosphorylation sites among different organisms, the human sequences from this database were used to query the proteomes of chicken, turkey, mouse, and chimpanzee using the DAPPLE software platform (15). This analysis provided us with both an indication of the relative similarities of kinase target sequences between each species and human, and a relative level of similarity between chicken and turkey within this portion of the proteome. As expected, the chimpanzee was most similar to human in phosphorylation site conservation, with 82.98% of the 15-mer queries being identical between human and chimpanzee (Table 2). This was a significantly greater percentage than mouse, chicken, or turkey, but it is much less than the sequence identity observed between the human and chimpanzee genomes (31). Of the four species analyzed, mouse was the next most similar to human beings, which was expected considering both are mammals. Finally, comparing human 15-mer peptides to chicken and turkey indicated that these two species are relatively similar in their distance from human: 16.20% of sequences exhibited complete identity in chicken vs. 15.15% in turkey. Conversely, approximately 40% of the human 15-mers had very weak matches (seven or more sequence differences) in both the chicken and turkey proteomes.

**Table 2:** **Conservation between human kinase target sequences and those of various other species**.

Sequence differences	Peptides vs. human (out of 146,765)			
	Chimpanzee (%)	Mouse (%)	Chicken (%)	Turkey (%)
0	82.98	36.67	16.20	15.15
1	7.21	17.41	10.29	9.74
2	1.38	11.34	8.63	8.33
3	0.64	7.77	7.31	7.26
4	0.51	5.71	6.27	6.09
5	0.59	4.45	5.85	5.72
6	0.96	4.11	7.41	7.35
7+	5.72	12.54	38.04	40.36

*Known human phosphorylation sites were obtained from PhosphoSitePlus (25) and were searched using DAPPLE (15) against each animal proteome. Sequences differences are out of the 15 total amino acids per sequence and % of peptides are the number of peptides out of the total analyzed that had the indicated number of differences within its 15-mer*.

Following the comparison of phosphorylation site conservation between human and each of mouse, chicken, and turkey, we compared the 15-mer sequences in chicken (as predicted by DAPPLE using all of the entries in PhosphoSitePlus as queries) to the turkey proteome, and vice-versa. In the chicken to turkey comparison, 69.19% of putative chicken phosphorylation sites had exact matches in the turkey proteome (Table 3). In the reverse comparison, 75.61% of turkey 15-mers had exact matches in the chicken proteome. The difference in percentage of exact matches can likely be attributed to the proteome sizes of the two species. Given that the chicken proteome (approximately 23,000 proteins) is significantly larger than the turkey proteome (approximately 16,000 proteins) (29), it was not unexpected that a higher percentage of turkey peptides would have exact matches in the chicken proteome compared to the reverse.

**Table 3:** **Conservation between chicken and turkey phosphorylation target sites**.

	Chicken to turkey	Turkey to chicken
	
Total peptide sequences	170,709	165,647

Sequence differences	% of peptides	% of peptides
0	69.19	75.61
1	10.20	10.94
2	3.68	3.31
3	1.90	1.55
4	1.37	1.06
5	1.30	1.00
6	1.78	1.23
7+	10.58	5.31

*Total peptide sequences refer to the total number of amino acid sequences within the first species’ proteome. Sequences differences are out of the 15 total amino acids per sequence and% of peptides are the number of peptides out of the total analyzed that had the indicated number of differences within its 15-mer*.

The level of phosphorylation site conservation between chicken and turkey suggests that it would be possible to design a single array that is specific to both chicken and turkey. Given the percentages reported in Table 2, there are tens of thousands of peptides that are found as exact matches in both the chicken and turkey proteomes, and thus that could potentially be selected for inclusion on an array that is equally applicable to chicken and turkey. Nonetheless, it is often desirable to include very specific peptides of interest on the array. When we consider that the genetic homology between these two species is approximately 90%, we see that there are significant differences between these two species at the proteome level that must be considered in any proteomic analysis. As such, it is not necessarily possible to create an array that is equally applicable to both turkey and chicken, as some of the kinase target sequences the researcher wants represented may have sequence differences between chicken and turkey.

**Figure 1:**
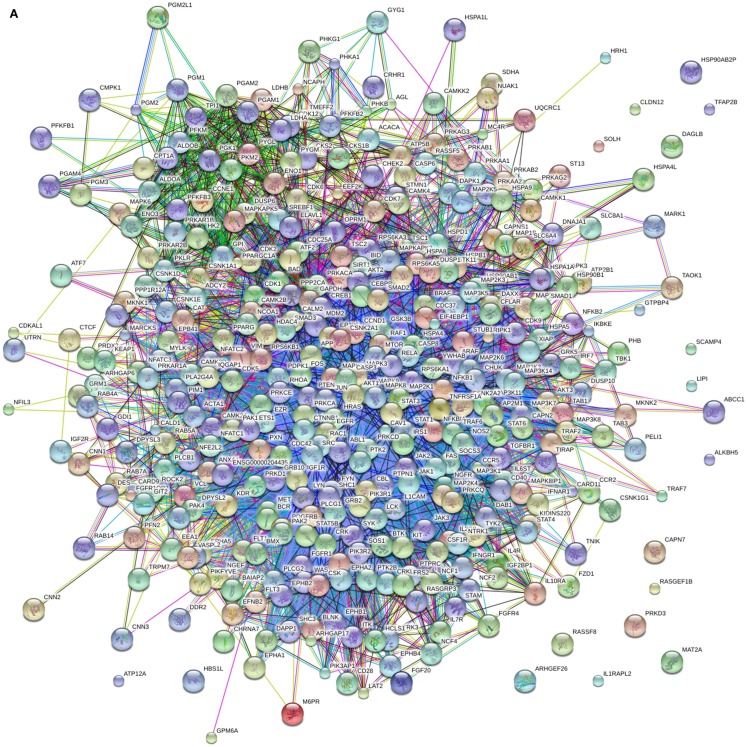

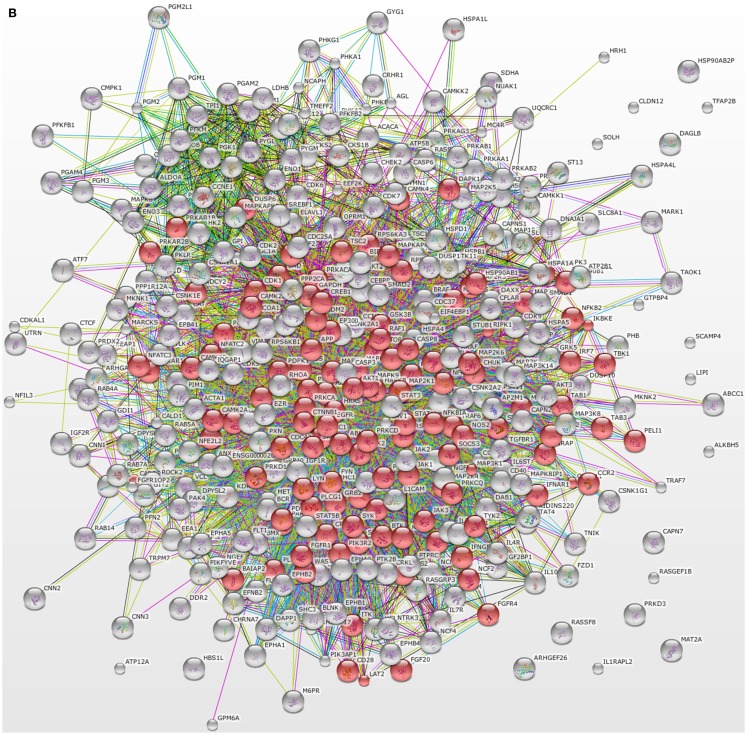

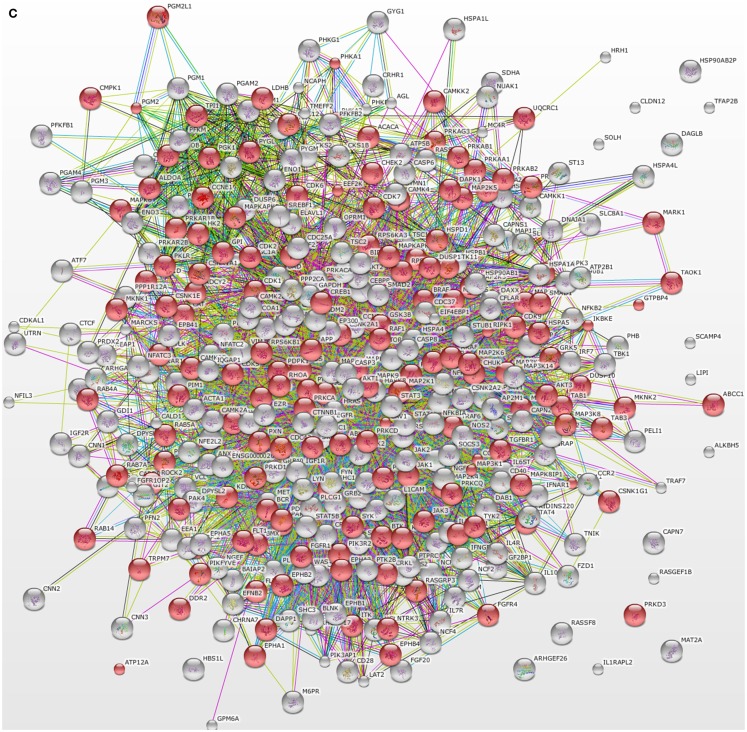
**STRING output of interactions between proteins of the original chicken immune and metabolic peptide arrays**. **(A)** Protein-protein interactions between proteins included in the immune peptide array and metabolic peptide array. **(B)** Red circles indicate immune-related proteins based on GO Biological Function terms **(C)** Red circles indicate metabolism-related proteins based on GO Biological Function terms. The figure illustrates the numerous interactions between the immune and metabolism related proteins.

We have previously used chicken-specific peptide arrays designed to study immune response (32) and metabolic changes (10). Using the STRING protein–protein interaction database (33)^4^, we looked into the potential links between proteins represented on these two functionally distinct arrays. Figure 1A shows the complete network of interactions generated from the unique proteins represented by the two arrays. Using the GO term Biological Process categorization feature of STRING, we were able to highlight the proteins within the network that corresponded to immune processes (Figure 1B) and metabolic processes (Figure 1C). These figures show that proteins from the two distinct biological process categories do not show up as distinct nodes (i.e., separated physically in the figure), but are interspersed and linked to one another in a number of ways. Based on the number of connections between the “immune” proteins and the “metabolism” proteins, it could be argued that these protein groupings are not distinct at all; thus, we have the rationale for the design of the first-ever species-specific kinome peptide array for the study of the integrated immuno-metabolomic responses within the two poultry species.

The ideal design for an immuno-metabolic array would incorporate as large a cross section of the two biological processes as possible, selected from the broad immune and metabolism categories. These include fatty acid, carbohydrate and protein metabolism, innate and adaptive immunity, hormonal metabolism and response, and key signaling molecules that cover the central signaling hubs of key pathways. Peptides representing proteins’ kinase target sites from these groups were individually selected from the DAPPLE output to be included on the combined immuno-metabolic array. Table 4 provides a breakdown of the number of peptides on the final array and the biological processes in which they are involved. Since many of the proteins on the array are involved in more than a single process, there are a large number of proteins listed within Table 4. The fact that many proteins are involved in several different metabolism- and immune-related biological processes emphasizes the numerous links between immune and metabolic signaling (as observed in Figure 1).

**Table 4:** **GO term biological processes incorporated into immuno-metabolic peptide array**.

GO ID^1^	Term description	No. of proteins	% of Total
GO:0019222	Regulation of metabolic process	166	42.56
GO:0031323	Regulation of cellular metabolic process	163	41.79
GO:0006796	Phosphate-containing compound metabolic process	158	40.51
GO:0080090	Regulation of primary metabolic process	158	40.51
GO:0006793	Phosphorus metabolic process	157	40.26
GO:0010033	Response to organic substance	156	40.00
GO:0006950	Response to stress	152	38.97
GO:0019538	Protein metabolic process	152	38.97
GO:0060255	Regulation of macromolecule metabolic process	150	38.46
GO:0051246	Regulation of protein metabolic process	143	36.67
GO:0071310	Cellular response to organic substance	139	35.64
GO:0044267	Cellular protein metabolic process	138	35.38
GO:0009893	Positive regulation of metabolic process	137	35.13
GO:0002376	Immune system process	130	33.33
GO:0010604	Positive regulation of macromolecule metabolic process	129	33.08
GO:0019220	Regulation of phosphate metabolic process	127	32.56
GO:0045087	Innate immune response	123	31.54
GO:0051171	Regulation of nitrogen compound metabolic process	121	31.03
GO:0010468	Regulation of gene expression	119	30.51
GO:0002682	Regulation of immune system process	112	28.72
GO:0032270	Positive regulation of cellular protein metabolic process	101	25.90
GO:0045937	Positive regulation of phosphate metabolic process	96	24.62
GO:0002764	Immune response-regulating signaling pathway	90	23.08
GO:0070848	Response to growth factor	82	21.03
GO:0009725	Response to hormone	78	20.00
GO:0033554	Cellular response to stress	78	20.00
GO:0044281	Small molecule metabolic process	77	19.74
GO:0002684	Positive regulation of immune system process	76	19.49
GO:0009891	Positive regulation of biosynthetic process	74	18.97
GO:0002768	Immune response-regulating cell surface receptor signaling pathway	72	18.46
GO:0050778	Positive regulation of immune response	72	18.46
GO:0009056	Catabolic process	70	17.95
GO:0002757	Immune response-activating signal transduction	67	17.18
GO:0002253	Activation of immune response	66	16.92
GO:0009892	Negative regulation of metabolic process	64	16.41
GO:0032870	Cellular response to hormone stimulus	61	15.64
GO:0045935	Positive regulation of nucleobase-containing compound metabolic process	61	15.64
GO:0043434	Response to peptide hormone	57	14.62
GO:0009894	Regulation of catabolic process	52	13.33
GO:0045088	Regulation of innate immune response	51	13.08
GO:0051254	Positive regulation of RNA metabolic process	50	12.82
GO:0032869	Cellular response to insulin stimulus	43	11.03
GO:0051248	Negative regulation of protein metabolic process	40	10.26
GO:0002758	Innate immune response-activating signal transduction	39	10.00
GO:0008286	Insulin receptor signaling pathway	39	10.00
GO:0045089	Positive regulation of innate immune response	39	10.00
GO:0006091	Generation of precursor metabolites and energy	38	9.74
GO:0002429	Immune response-activating cell surface receptor signaling pathway	37	9.49
GO:0032269	Negative regulation of cellular protein metabolic process	35	8.97
GO:0002520	Immune system development	35	8.97
GO:0006954	Inflammatory response	33	8.46
GO:0080135	Regulation of cellular response to stress	32	8.21
GO:0006006	Glucose metabolic process	31	7.95
GO:0009617	Response to bacterium	31	7.95
GO:0048545	Response to steroid hormone	30	7.69
GO:0019318	Hexose metabolic process	28	7.18
GO:0005996	Monosaccharide metabolic process	28	7.18
GO:0019216	Regulation of lipid metabolic process	28	7.18
GO:0031667	Response to nutrient levels	24	6.15
GO:0016052	Carbohydrate catabolic process	21	5.38
GO:0002433	Immune response-regulating cell surface receptor signaling pathway involved in phagocytosis	21	5.38
GO:0060191	Regulation of lipase activity	20	5.13
GO:0009743	Response to carbohydrate	20	5.13
GO:0016051	Carbohydrate biosynthetic process	18	4.62
GO:0002819	Regulation of adaptive immune response	17	4.36
GO:0050864	Regulation of B cell activation	15	3.85
GO:0019217	Regulation of fatty acid metabolic process	14	3.59
GO:0006094	Gluconeogenesis	12	3.08
GO:0019319	Hexose biosynthetic process	12	3.08
GO:0046364	Monosaccharide biosynthetic process	12	3.08
GO:0060396	Growth hormone receptor signaling pathway	11	2.82
GO:0071378	Cellular response to growth hormone stimulus	11	2.82
GO:0005977	Glycogen metabolic process	11	2.82
GO:0044042	Glucan metabolic process	11	2.82
GO:0006073	Cellular glucan metabolic process	11	2.82
GO:0005980	Glycogen catabolic process	10	2.56
GO:0009251	Glucan catabolic process	10	2.56
GO:0044247	Cellular polysaccharide catabolic process	10	2.56
GO:0000272	Polysaccharide catabolic process	10	2.56
GO:0044275	Cellular carbohydrate catabolic process	10	2.56
GO:0006096	Glycolysis	10	2.56
GO:0060334	Regulation of interferon-gamma-mediated signaling pathway	9	2.31

*GO Term Biological Processes incorporated into immuno-metabolic peptide array. Table outlines the relative protein composition of the chicken and turkey immuno-metabolic peptide arrays, equivalent proteins were chosen for each species. # of Proteins indicates the number of proteins on the array that fit into the given biological process, % of total is the percentage of total peptides on the array represented by this given biological processes. The STRING online database placed the given number of proteins into their GO Term categories following input of the peptide array protein list*.

*^1^1GO ID refers to the identification number for the Biological Processes with the Gene Ontology Database. Further information of the given biological process can be found by querying the databse using the GO ID number*.

As the proteome analysis indicated, significant differences in the proteomes of chicken and turkey, and specifically within the kinase recognition sites of phosphorylated proteins, suggest that distinct chicken and turkey peptide arrays should be designed. With the level of proteome divergence, even within these evolutionarily close species, a cross-species chicken/turkey array would not take full advantage of the potential for maximum kinase recognition and signal detection possible with a species-specific array. Table S1 in Supplementary Material includes the proteins, peptide sequences, accession numbers, and protein annotations for the chicken immuno-metabolic peptide array. Table S2 in Supplementary Material includes the DAPPLE output for the equivalent proteins within the turkey.

## Discussion

In general, genome sequencing and genetics have highlighted the remarkable similarity across species and have emphasized the evolutionary links among different species. However, logically, considering the huge level of phenotypic variation between species, even within the mammalian or avian classes, we must assume this diversity comes from distinctions at the protein level. Our analysis here, and elsewhere (6), has emphasized the diversity found between the proteomes of various species, specifically at the level of the phosphoproteome, which is our focus. Chicken and turkey are two relatively closely related species; their genome similarities exhibit this despite approximately 40 million years of species divergence (21). However, our protein-to-protein comparison showed that there is greater divergence at the protein level, as even orthologous proteins between the two species were on average only 83.28% identical. This result alone highlights what poultry researchers, based on their own first-hand experience, have known: turkeys are not simply “big chickens.”

The level of distinction in the proteome is also observed within the phosphorylation target sites recognized by kinases. In a phosphosite-to-phosphosite comparison, which represents well over 200,000 phosphorylation target sequences, approximately 70–75% were found as exact matches in both the chicken and turkey proteomes (Table 3). In comparison, when comparing the human peptide sequences to that of our closest evolutionary relative, the chimpanzee, 82.98% were identical in both proteomes. This analysis suggests that using chicken as a model for the turkey may, in relative terms, be less valid than using chimp as a model for human. The level of divergence at these important enzymatic sites emphasizes the need for a turkey-specific approach to the study of this species. If this is the case for turkey and chicken, it is worth noting that between humans and mice the differences are more pronounced; there is only complete phosphorylation site sequence identity in 36.67% of peptides. Considering the sheer number of studies that utilize the mouse as a model for human physiology, it is worth taking into account that these species diverge so significantly at the phosphoproteome level. These differences highlight the need and the potential power of designing species-specific peptide arrays to study the kinomes of even closely related species.

We have described here three levels of comparison between the chicken and turkey: (1) genome, (2) orthologous protein, and (3) kinase recognition target site. At each of these levels, the relative similarity between chicken and turkey diminishes. While the genome level displays nearly 90% identity the kinase target sequence identity is between 70 and 75%. This indicates that the phenotypic species divergence is greater at the proteome level and even greater still at this site of enzymatic activity.

Our group has conducted numerous studies on the physiology and specifically the host–pathogen interactions of several agricultural species (including chicken) using the species-specific peptide array technique. To date, we have focused on individual biological processes, such as metabolism or innate immunity. Our analysis here shows that the metabolism and immunity processes may be distinctions without a difference. Figure 1 highlights the interactions that are present between proteins that are part of these two putatively different biological functional groupings. This level of interaction was the impetus for our design of an immuno-metabolic, species-specific peptide array for chicken and turkey that we report here for the first time. In the realm of animal agriculture, nutrition/metabolism and immune performance have been two fields that have been converging for many years. Producers, veterinarians, and animal science researchers have come to understand that a sole focus on growth can often come at the expense of health and disease susceptibility, and a strong response to disease can have significant effects on growth (24). With a tool that can study both metabolism and immunity simultaneously, these two areas of animal science (and poultry production) no longer have to be at odds. We can study nutrition and observe effects on immune responses or, conversely, study disease and see how this may effect growth. Our group has already shown that a *Salmonella* infection of chicken can have effects on the fat deposition and carbohydrate metabolism in peripheral muscle (10). We look forward to the discovery that will be possible when the tool we have described is put to use on any number of nutritional-, metabolic-, and disease-related questions affecting the poultry industry and poultry health.

## Author Contributions

Ryan J. Arsenault conceived of the study, set out the analysis parameters, designed the final immuno-metabolic array, and wrote the paper. Brett Trost conducted the proteome comparisons and generated most of the data outputs. Michael H. Kogut advised on analysis presentation and provided experimental resources. Ryan J. Arsenault and Brett Trost analyzed the data. Brett Trost and Michael H. Kogut reviewed and edited the paper.

## Conflict of Interest Statement

The authors declare that the research was conducted in the absence of any commercial or financial relationships that could be construed as a potential conflict of interest.

## Supplementary Material

The Supplementary Material for this article can be found online at http://www.frontiersin.org/Journal/10.3389/fvets.2014.00022/abstract

Supplemental Table S1 **Chicken immuno-metabolism design table**. The table includes the human proteins and their chicken equivalents, along with the chicken-specific kinase target peptide sequences and the human annotations of the proteins.

Supplemental Table S2 **Turkey DAPPLE output of the equivalent proteins as on the chicken array**. The table includes the DAPPLE output file for the turkey proteins to be included in the turkey-specific immuno-metabolism peptide array. Each protein to be incorporated into the array contains numerous peptide sequences which were Blast hits within the turkey proteome. These individual sequences are then selected for incorporation onto the peptide array. “Query” represents annotated database species and “Hit” represents species of interest: turkey.

## Footnotes

^1^http://saphire.usask.ca/saphire/dapple/index.html

^2^http://www.phosphosite.org

^3^http://cegg.unige.ch/orthodb6

^4^http://string-db.org
